# Supplementary material for: Identification of photoperiod-induced specific miRNAs in the adrenal glands of Sunite sheep (Ovis aries)
Source: Front Vet Sci. 2022 Jul 22;9:888207. doi: 10.3389/fvets.2022.888207 (PMC9354845; doi:10.3389/fvets.2022.888207)
Supplement: Supplementary file 1 [file Data_Sheet_1.ZIP › Supplementary file/miRDeep2 file.zip!/miRDeep2 file/Know_Structure/LP42A1/oar-mir-19b.pdf]

miRBase precursor : oar-mir-19b
Total read count : 2851
oar-miR-19b read count : 2840
remaining reads : 11

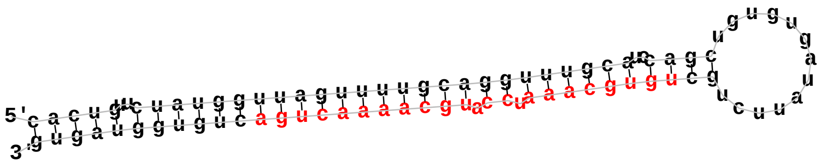

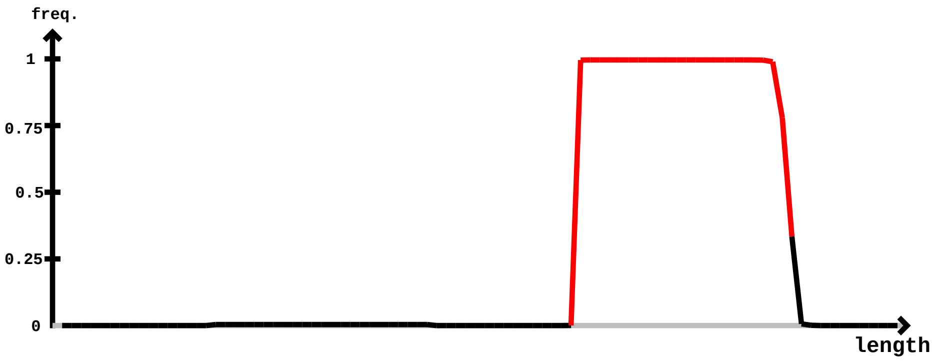

oar-miR-19b

```
5'- cacuguucuaugguuaguuuugcagguuugcauccagcugugugauauucugcugugcaaauccaugcaaaacugacugugguagug   -3'    exp
    (((((..(((((((((((((((((((((((((..((((.............))))))))))).)).))))))))))))))))))))))    reads      mm          sample
    ...............aguuuugcagguuugcauccagc.................................................    11         0           seq
    ....................................................cugugcaaauccaugcaaaacug............    1          0           seq
    .....................................................ugugAaaauccaugcaaa................    1          1           seq
    .....................................................ugugcaaauccaugcaaaa...............    1          0           seq
    .....................................................ugugcaaauccaugcaaaac..............    16         0           seq
    .....................................................ugugcaaaucUaugcaaaac..............    2          1           seq
    .....................................................ugugcaaaucUaugcaaaacu.............    51         1           seq
    .....................................................ugugcaaauccaugcaaaacu.............    547        0           seq
    .....................................................ugugcaaaucUaugcaaaacug............    105        1           seq
    .....................................................ugugcaaauccaugcaaaacug............    1163       0           seq
    .....................................................ugugcaaauccaugcaaaacuga...........    800        0           seq
    .....................................................ugugcaaaucUaugcaaaacuga...........    133        1           seq
    .....................................................ugugcaaauccaugcaaaacugaU..........    13         1           seq
    .....................................................ugugcaaauccaugcaaaacugac..........    2          0           seq
    .....................................................ugugcaaauccaugcaaaacugacu.........    1          0           seq
    .....................................................ugugcaaauccaugcaaaacugaUu.........    2          1           seq
    ......................................................gugcaaauccaugcaaaacu.............    1          0           seq
    ......................................................gugcaaauccaugcaaaacug............    1          0           seq
```
